# Mushroom mycelia as sustainable alternative proteins for the production of hybrid cell‐cultured meat: A review

**DOI:** 10.1111/1750-3841.70060

**Published:** 2025-02-07

**Authors:** Kayise Hypercia Maseko, Thierry Regnier, Paul Bartels, Belinda Meiring

**Affiliations:** ^1^ Department of Biotechnology and Food Technology Tshwane University of Technology Pretoria Republic of South Africa; ^2^ WildBio Company Pretoria Republic of South Africa

**Keywords:** cultivation, culture media parameters, fermentation, microbial proteins

## Abstract

World agriculture endures an immense challenge in feeding the world's growing population in the face of several productivity and environmental threats. Yet, the demand for alternative protein sources is rapidly increasing as a result of population growth, including health and ethical concerns associated with meat consumption. Edible mushroom species contain a high composition of protein, fiber, vitamins, and a variety of minerals, and are regarded as sufficient sources of food products. *Pleurotus* genus is one of the most extensively studied edible fungi due to its exceptional physical, chemical, biological, and enzymatic properties. The assessment on the effects of the in vitro culture media composition, including carbon and nitrogen sources, pH, and temperature are all necessary for enhancing mushroom mycelial biomass growth and production. Mycoprotein as a fungal‐derived protein source has been identified as a more sustainable and healthier meat substitute due to its fibrous structure, high nutritional value, and unique functional profile. Its distinctive production method results in a much lower carbon and water footprint than traditional farming methods. A systemic transition from traditional agriculture to more sustainable cellular agriculture using cell‐cultivation methods to create animal products has been proposed and initiated. This review can provide an overview on the various processes involved in the production and usage of mycelium as an alternative protein source in hybrid cell‐cultured meat production.

## 1 INTRODUCTION

The connection between increasing population, climate change, food ethics, and health is multifactorial. Higher population growth increases food production, emissions, and vulnerability to climate‐related and health effects (Derbyshire, 2020). The lengthy process of breeding, rearing, and butchering of whole livestock for meat production involves the consumption of a lot of water, land, and the usage of fuel energy, causing substantial environmental pollution (Guan et al., 2021). Consecutively, the negative impact of traditional meat‐related ethical issues and foodborne illnesses has gained a lot of attention in the global sector, with concerns on how to mitigate it (Rischer et al., 2020).

It has been reported that by 2050, the eight billion people who currently live on the planet will have surpassed 10 billion. Given the limited resources and cultivatable land, it will be very difficult since, according to the Food and Agriculture Organization (FAO) 70% more food would be expected in 2050 to sustain the necessities of the growing population (Bonny et al., 2017). Despite the vast range of economic, environmental, cultural, and social services that livestock farming provides at the local, regional, and international levels, a substantial number of cattle are currently farmed using the factory farming model. Animal farming will be expected to produce more premium and affordable animal‐derived products through sustainable, ethical, and economically viable production systems (Chriki & Hocquette, 2020).

Not only is the demand for more food critical, but alternative protein sources have also seen an increase in demand in recent years. A variety of causes are driving protein demand, including the link to the health benefits of eating meat, the high demand of dairy, and other animal proteins due to diet supplements and food consumption. There has also been a rise of vegan, vegetarian, and flexitarian populations that has accelerated the use of plant proteins in food items. Overall, the developing food sector is driving the protein market and the demand for alternative protein components, owing to rising population and consumer awareness (Ismail et al., 2020). To address the challenges of producing more food for a growing world population, especially if alternative proteins are part of the solution, accessibility and affordability are critical components (Wood & Tavan, 2022).

Agriculture, on the other hand, accounts for 70–85% of the global water footprint and 30% of greenhouse gas emissions globally (Smetana et al., 2015). In which the irrigation of cattle feed crops accounts for about 8% of global human water use (Bhat et al., 2020). Additionally, agricultural intensification and food production has a detrimental impact on soil and biodiversity, with a negative effect on food security and on‐farm revenue at local levels (Mbow et al., 2014). As a result, reducing the environmental footprint is thus a major reason for considering industrial biotechnology of plants used for food applications. Animal agriculture can be geared on more sustainable systems, using controlled methods and advanced techniques limiting environmental impacts (Jurgilevich et al., 2016; Rischer et al., 2020).

The detrimental impact of traditional animal farming can be minimized by the adoption of numerous habits, such as dietary changes including the consumption of alternative protein sources. Sustainable diets are those that integrate three key elements: environmental, social, and economic. Infrastructures and technology, as well as inhabitants' competencies, practices, and world views, must shift to make the transition to sustainability and food security (Jurgilevich et al., 2016; Rust et al., 2020). Edible mushroom mycelia, rich in proteins, nutrients, and bioactive components, are often utilized as nutritional supplements or health care products in the food and pharmaceutical industries. These mycelia can be produced in vitro with minimal environmental pollution and footprint (Xv et al., 2024). The potential application of mycelia as a meat alternative or substitute is gaining interest due to their simple culture methods, nutritional value, fibrous texture, and alignment with growing consumer demands for sustainable and healthier food options (Finnigan et al., 2024). Currently, limited research has been conducted on the functionalities and applications of blending mycelia with plant and animal proteins to produce hybrid‐cell‐cultured meat products. Despite this, the field is still emerging, requiring further studies to fully explore and optimize the structural aspects that cell‐cultured meat may lack (Santhapur et al., 2024). Given the challenges of achieving texture requirements while enhancing the flavor and nutritional profile of cultured meat, fungi present a viable alternative source. Additionally, considerations must be given to the scalability challenges of hybrid food systems, such as the supply chain issues related to maintaining a reliable raw material supply, and regulatory approvals that delay market entry of new products (Kumar et al., 2023).

## 2 MUSHROOM MYCELIA: A FOOD SOURCE

Mushrooms are a specialized type of fungus that represent fruiting bodies with a wide range of shapes, sizes, colors, and textures. Various species are located all over the world, some of which are particularly specific to some geographic areas, while others are universal and can thrive in different seasons within the same region (Halbwachs et al., 2016). Edible mushrooms are commonly produced for commercial purposes and utilized as part of the human diet (El‐Ramady et al., 2022). However, postharvest processing of mushrooms is another barrier to commercial appeal and nutritional value preservation. Due to their high perishability, a shorter shelf life (two to three days) is achieved at ambient temperature (Sande et al., 2019), whereas their high water content makes them susceptible to spoilage, microbial growth, and enzymatic browning (Akbarirad et al., 2013). As an alternative to macrofungi, mushroom mycelium is easier to cultivate and produce (Stoffel et al., 2019), with production reported to have begun in 1949 as an alternative to mushrooms for culinary purposes (Chahal, 1989). This is mainly since fungal proteins are thought of as a vegan protein source since they include all the essential amino acids, including umami amino acids giving them that meaty taste (Bakratsas et al., 2021).

### 2.1 Nutritional composition

Edible fungi rank third in terms of food sources, after plant‐ and animal‐based meals, and provide nearly all the nutrients required for the daily diet. They are rich in minerals and amino acids in addition to abundant amounts of protein, cellulose, and polysaccharides (Yu et al., 2020). The amount of fat available in fungi is low (1% to 6%), making it a low‐calorie food (Niego et al., 2021). Among its many medical uses are the enhancement of human immunity, anticancer activity, and blood lipid reduction. Along with other medical uses, they aid in decreasing blood cholesterol and enhancing human immunity (Yu et al., 2020).

With the increased need for alternative food sources that are nutritious, scalable, affordable, and environmentally sustainable (Holt et al., 2024), mycelium has gained a lot of attention since its nutritional makeup can be replicated to that of mushroom fruiting bodies (Rathore et al., 2019). Table 1 compares the nutritional makeup of mushrooms and mycelial biomass, which might vary depending on the species and substrate or culture conditions. These nutrients can be beneficial to individuals who only consume plant‐based meals (Holt et al., 2024). Despite this, the levels of heavy metals available in mushroom fruiting bodies (owing to the presence of metal fractions in the soil) can be accumulated by mycelium during growth by supplementing the culture media with a metal salt to complement potential deficits in human nutrition (Berger et al., 2022).

**TABLE 1:** Comparative nutritional composition of mushrooms and their mycelium.

Nutritional composition	Mushroom fruiting body^a^	Mycelium biomass^a^	References
**Protein**	Contains protein levels ranging from 10% to 48%, dwb.	Certain filamentous fungi have been identified to contain more protein (up to 85% dwb.) than most mushrooms, making them viable alternative protein sources (to animal protein).	Berger et al. (2022), Souza Filho et al. (2018)
**Amino acids**	Contains alanine, arginine, aspartic acid, glutamic acid, leucine, lysine, phenylalanine, serine, proline, threonine, tyrosine, and valine.	Contain all the essential amino acids, including cysteine, methionine, and ergothioneine (a potent antioxidant and bioactive amino acid).	Barakat and Sadik (2014), Rathore et al. (2019)
**Vitamins and minerals**	The accumulation of heavy metals, including radionuclides, in fruiting bodies is widely recognized, whereas they are a good source of B vitamins (B_1_, B_2_, B_3_, B_5_, B_6_).	Mycelium accumulates minerals from the culture medium source. While reported to supply essential micronutrients (copper, folate, iron, niacin, riboflavin, vitamin B_12_, and zinc) in the diet, accounting for at least 20% of the daily value. Also contains B vitamins, especially during active growth.	Berger et al. (2022), Holt et al. (2024)
**Carbohydrates**	Rich in the complex carbohydrate's chitin and β‐glucans. Contain disaccharides and oligosaccharides including sucrose, maltose, xylose, rhamnose, mannose, and fructose. Mannitol is the most abundant sugar, accounting for approximately 80% of total free sugars.	Lower carbohydrate content due to mycelium prioritizing protein synthesis. Low in β‐glucans due to thin, porous structure for nutrient absorption. Contains the simplest form of polysaccharide, mainly glucose	Berger et al. (2022), Das and Prakash (2022), Niego et al. (2021), Olivero et al. (2023)
**Fatty acids**	Contain saturated and unsaturated fatty acids (linolenic acid, oleic, and palmitic acid). Oleic and palmitic acids are most identified with Basidiomycota phylum.	Low in total fat (mainly unsaturated fatty acids).	Niego et al. (2021), Sande et al. (2019), Holt et al. (2024)

^a^ May vary according to the fungi species, strain, and cultivation conditions. Dry weight basis—dwb.

The most accepted and cultivated edible mushroom species worldwide include *Agaricus bisporus*, *Lentinula edodes*, and *Pleurotus spp*. (particularly oyster mushroom) (Bakratsas et al., 2021; Niego et al., 2021). This is mainly due to their high concentration of proteins, vitamins, and minerals, making them excellent nutritional foods (Bakratsas et al., 2021).

**FIGURE 1:**
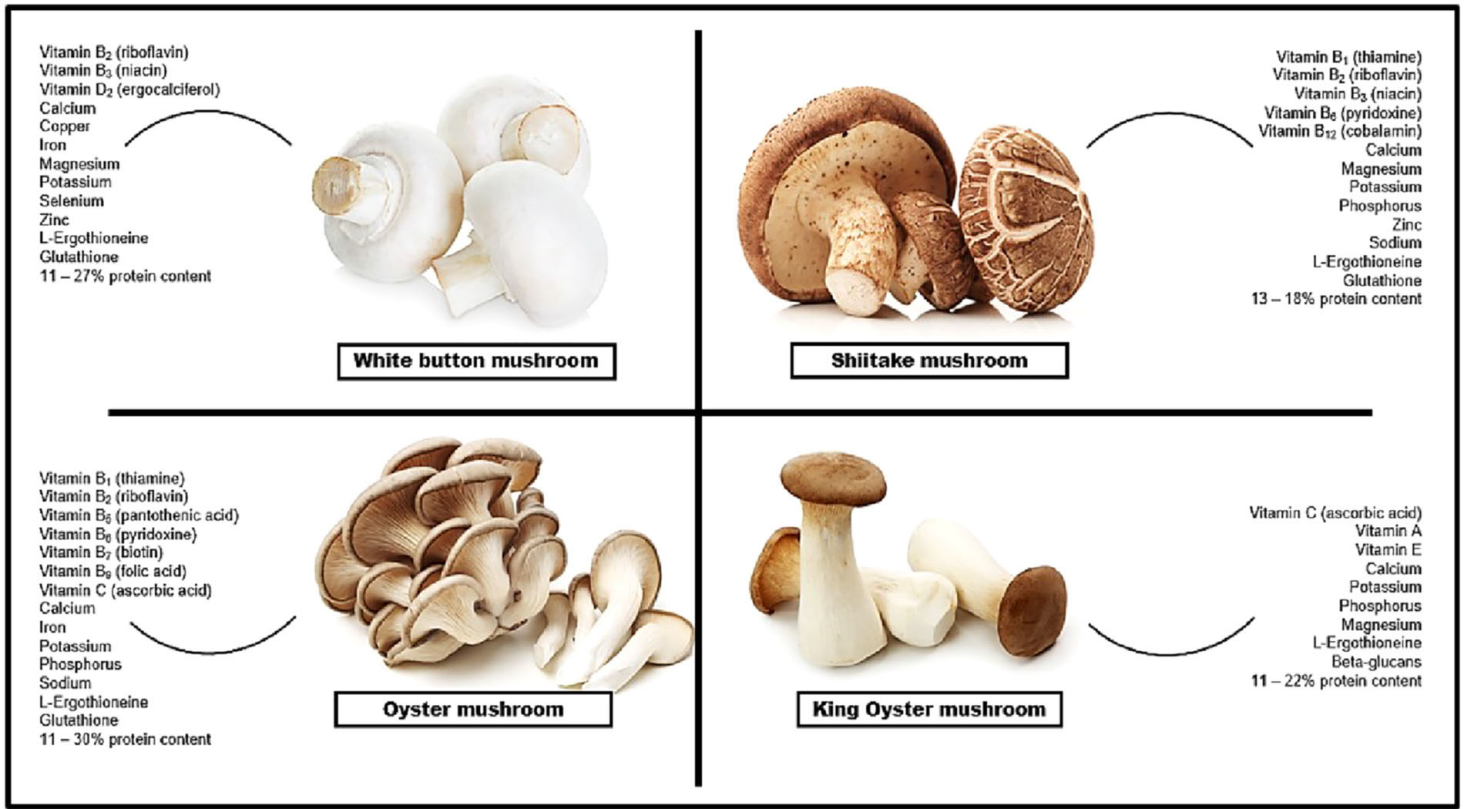
The nutritional composition of four mushroom species: white button, shiitake, oyster, and king oyster mushrooms fruiting body. The protein content of these species ranges from 11% to 30% demonstrating their potential as significant sources of alternative protein. (© Images courtesy of Chengyu Zheng, Egal, and Yv Davyd, obtained from iStock: https://www.istockphoto.com/).

The nutritional composition of the white button (*A*. *bisporus*) mushroom, shiitake (*L*. *edodes*) mushroom, oyster (*P*. *ostreatus*) mushroom and king oyster (*P*. *eryngii*) mushroom is displayed in Figure 1. The inclusion of these nutrient‐dense mushrooms in meat formulations could substantially enhance the nutritional profile, flavor, and overall health benefits of the products. This underscores the viability of utilizing these mushrooms as sustainable and nutritious ingredients in the development of meat products (Majumder et al., 2024). Whereby the mycelium counterparts have been widely reported for their composition with similar values (Berger et al., 2022).

Additionally, multiple studies have revealed that various mushroom species are beneficial in both the prevention and treatment of a variety of chronic diseases, including cancer, cardiovascular disease, diabetes mellitus, and neurodegenerative diseases. It can be inferred that including mushrooms and mycelium in one's regular diet could be a natural adjuvant in the treatment and prevention of a variety of chronic diseases (Roncero‐Ramos & Delgado‐Andrade, 2017).

### 2.2 Morphology, structure, and potential application

**FIGURE 2:**
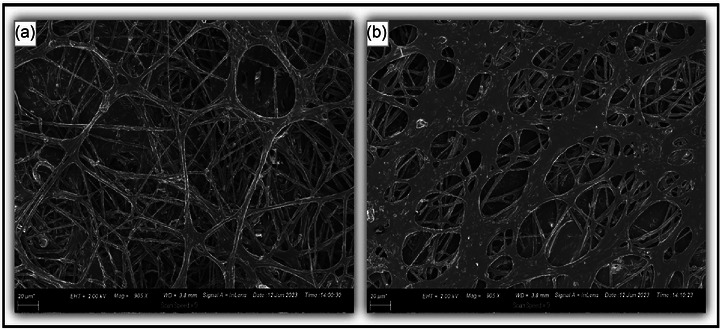
Scanning electron microscope (SEM) images of various carbon‐coated hyphal morphologies of lyophilized filamentous Pleurotus sp. mycelium structures relevant to meat applications. (a) Mycelium cultivated on hemp seed‐based medium, exhibiting a dense and intricate fibrous network. (b) Mycelium cultivated on marula seed‐based medium, displaying a more open and porous structure.

Mycelium, the root‐like part of fungi, is filamentous (white thin strands) that develops and fuse to form a mass of branching hyphae (Derbyshire, 2020). This is seen in Figure 2, displaying a network of interconnected hyphae of varying thickness.

The dense structure observed in Figure 2a is indicative of a stronger, more cohesive matrix, which is essential for creating a meat‐like texture and mouthfeel of conventional meat. Conversely, the more porous network in Figure 2b may contribute to a different mouthfeel and moisture retention, which are both critical factors in the sensory attributes of alternative meat products (Akromah et al., 2023).

In submerged agitated liquid media, the fungal spores entangle and form spherical mycelium owing to the liquid's shear force. On the other hand, when the fungi are cultivated in solid medium, the mycelium will grow evenly spread flat, covering the surface of the medium, displaying a 2D planar structure, and the capability to colonize larger areas, creating a fluffy or compact layer known as “fungal skin” (Ghazvinian et al., 2019; Xia et al., 2022).

The primary components of the fungal cell wall include chitin (principal fibrous polysaccharide present in fungi contributing to rigidity), β‐glucan (β‐D‐glucose polysaccharides), and glycoproteins (mannose and hydrophobins). The inner layer consists of chitin microfibrils, which are covalently cross‐linked with other polysaccharides, whereas the outermost layer is rich in glucans acting as mucilage (Manan et al., 2021).

Barzee et al. (2021) reported that pellet diameters can vary from less than 1 mm to a few centimeters (cm). Hyphae (fungal filaments) typically have cross‐sectional diameters of 3–5 µm and lengths of 400 – 700 µm, as reported by Okeudo‐Cogan et al. (2023). On the other hand, Antinori et al. (2021) reported that *Pleurotus* hyphae are typically larger, measuring on average 1.5 ± 0.4 µm, whereas *Ganoderma* hyphae are 0.7 ± 0.2 µm (thread‐like). Wherein such fibrous network configurations and diameters have been observed to be favorable for the development of superior meat substitutes or cell attachment (Barzee et al., 2021).

*Aspergillus oryzae*, *Fusarium venenatum*, *Fusarium* strain flavolapis, *Neurospora crassa*, and *Lentinula edodes* are among the species whose usage as a food source for mycelium has drawn attention from scientists and the food industry (Holt et al., 2024). On the other hand, mycelial species with fast growth rates, good texture, and flavor profiles like *Pleurotus* sp. (oyster mushroom) are currently being promoted for commercialization. These species have the potential to be cultivated in an environmentally friendly and sustainable manner, setting them up for future trends (Cheng et al., 2023).

Overall, the comprehensive examination of the nutritional composition of the various strains of mushroom mycelia, *Pleurotus* sp. unique morphology and structure that is primarily influenced by the culture conditions, and versatile potential applications highlights its value as a nutritious food source capable of meeting diverse dietary requirements.

## 3 MYCELIUM PRODUCTION: REVOLUTIONIZING AGRICULTURE

Given their naturally high secretory capacity, fungi are currently powerful hosts for producing high‐quality protein for food and other applications (Maini Rekdal et al., 2024). Their production can be accomplished without the need for much space or energy, in a controlled environment, and using various sources of culture media (Berger et al., 2022).

### 3.1 Cultivation using culture media

Culture media or growth media is classified into three types: submerged liquid fermentation, solid‐state fermentation, or semisolid‐state fermentation, which is based on the physical state (Gkerekou et al., 2021). In the laboratory, mycelia are usually cultivated in a sterilized nutritional medium enclosed in flasks (Brazkova et al., 2022). In industrial applications, mycelium is cultivated in sterile bioreactors (closed vessels) containing nutritional medium (Ouedraogo & Tsang, 2021). In addition, culture media recycling has received more attention as it relates to culture techniques, which has been successfully tested in bacterial and algal cultures (Lowrey et al., 2016) and mycelia recultivation (Jasińska, 2018; Sánchez, 2004), with promising results in terms of cost‐saving and sustainability.

The composition of culture media can be natural or synthetic. Natural culture media is prepared from varying concentrations of natural substrates such as herbaceous or woody stems, seeds, leaves, corn starch, wheat germ, rice straw, and oatmeal. Synthetic media, on the other hand, contain components that can be precisely replicated when prepared, with predetermined amounts of nutritional and nitrogen sources (Basu et al., 2015).

Fermentation is the production of mycelia in a type of medium containing dissolved nutrients, in which physical (temperature, aeration, agitation), chemical (pH, medium composition), and biological (inoculum, morphology, and rheology) parameters are controlled (Bakratsas et al., 2021). The specific culture conditions all have an impact on mycelial growth rate and high biomass production, as presented in Table 2. Moreover, the optimization of culture composition is essential for improving the mycelial biomass of microbial fermentation, which includes the carbon sources, nutritional, and nitrogen sources (Singh et al., 2020).

**TABLE 2:** Effect of the various culture medium factors and conditions on mycelial growth and production.

Culture medium			Effect on mycelial growth and production^a^	References
Factor	Type	Condition		
**Cultivation method**	Solid‐state culture medium	N/A	▪ Promotes the high productivity of mycelia in a stationery solid nutrient source, with the absence or near absence of free water.	Ouedraogo and Tsang (2021)
	Semisolid‐state culture medium	N/A	▪ The free liquid level is slightly increased to enhance nutrient availability for mycelium and mycelial fermentation control.	Machado et al. (2013)
	Submerged liquid culture medium	N/A	▪ Promotes mycelial productivity, yield potential, and lower possibility of contamination. Controlled agitation speed results in a balance between oxygen transfer and shear stress into the medium.	Kapoor et al. (2016)
**Culture** **process**	Culture medium	Aerobic	▪ The condition supports mycelial respiration and cell growth through the provision of dissolved oxygen in bioreactors as required, whereas flasks with media are continuously stirred to reinforce oxygen supply.	Bakratsas et al. (2021)
		Anaerobic	▪ They can cause impairment of normal respiratory activities leading to reduced production of biomass and bioactive compounds.	
**Culture temperature**	Culture house/incubator	≤18°C	▪ Lower temperatures result in a slower pace of cellular development and product synthesis	Bakratsas et al. (2021)
		22–25°C	▪ Results in optimal mycelial development, morphological characteristics, and higher yield potential.	Bellettini et al. (2019)
		≥25°C	▪ Temperatures over the optimal level can be lethal or have a detrimental impact on protein expression and metabolite synthesis, resulting in thermal death.	
**Illumination**	Culture house/incubator	Lightness	▪ Bright light treatment inhibits mycelial growth and cellular processes but promotes sporulation.	Zhang et al. (2023)
		Darkness	▪ Darkness is preferred to promote the rapid growth of mycelium, since fungi, as a heterotrophic organism, is not dependent on light to grow.	Karana et al. (2018)
**Incubation period**	Culture medium	≤7 days	▪ Mycelia is at its initial growth phase with the highest growth rate, differently impacted by the culture medium and method.	Kim and Kim (2009)
		8–15 days	▪ Mycelium further colonizes the medium, resulting in a thicker hyphal network. Harvested mycelium possesses the highest biomass weight, yield potential, and bioactive compounds.	
		≥20 days	▪ Excessive fermentative periods result in cell lysis, in which the cell wall may rupture or dissolve, whereby nutrients are depleted.	Wu et al. (2003)
**Inoculum** **type**	Liquid spawn	2% (v/v)	▪ Results in uniform mycelial biomass, optimal growth, and yield, which is influenced by the strain variability and the culture period.	Bakratsas et al. (2021)
	Agar plugs spawn	4% (w/v)	▪ Optimal biomass growth and development, whereas producing mycelial biomass of varying weight.	Martínez et al. (2023)
	Grain/seed spawn	N/A	▪ The spawn takes longer to grow while increasing the possibility of the culture medium and mycelium biomass contamination.	Abdullah et al. (2013)
**Moisture content**	Culture medium	Low levels	▪ Result in the inhibition of hyphae development.	Bellettini et al. (2019)
		Optimal levels	▪ Continuous moisture flow aids in the transportation of nutrients from the hyphae to the mycelial biomass.	
		High levels	▪ Results in the mycelium experiencing breathing difficulty, preventing perspiration, and rendering biomass development impossible.	
**pH level**	Culture medium	≤pH 4	▪ Has a negative effect on fungal cell membrane function, cell shape, and structure, therefore inhibiting growth.	Bakratsas et al. (2021)
		pH 5–pH 7	▪ Optimal mycelium growth and yields have been reported at these levels, which are crucial in cell growth and metabolite synthesis.	
		≥pH 8	▪ Negatively affects medium nutrient availability and mycelial enzyme activity, consequently inhibiting its growth.	
**Sterilization treatment**	Culture medium	121°C for 15 min	▪ Results in high‐quality mycelial production.	Lu et al. (2020)

^a^ May vary according to the fungi species and strain. Not applicable—N/A.

#### 3.1.1 Carbon sources

Carbohydrates are a major nutritional requirement for higher fungus growth and development since they are a fundamental component of the cytoskeleton (Xiao et al., 2006). The carbon sources: arabinose, dextrose, lactose, galactose, glucose, fructose, maltose, mannose, molasses, saccharose, sorbose, sorbitol, sucrose, xylose, and starch have all been used as supplements in the cultivation of mycelia. These carbon sources, which serve important structural and storage functions in the cell, are utilized differently among fungal species (Barakat & Sadik, 2014; Deshaware et al., 2021; Kirsch et al., 2016; Kumar et al., 2018).

Pereima and Ivanova (2017) study determined that glucose, sucrose, and molasses as the most suitable carbon sources for the cultivation of *Pleurotus* spp. Kirsch et al. (2016) determined that saccharose, fructose, and maltose were the three carbon sources that promoted the most considerable growth for *Pleurotus* spp. However, specific studies by Bakratsas et al. (2023) and de Andrade et al. (2021) on *P. ostreatus* and *P. eryngii* determined that glucose as a carbon source promoted the most considerable growth.

#### 3.1.2 Nutrient and nitrogen sources

Different fungal species have various nutritional requirements, whereby nutrient sources influence the formation of high‐density and vigor mycelium. Hence, the optimization of culture conditions is additionally important (Abdullah et al., 2013; Barseghyan et al., 2011). Pereima and Ivanova's (2017) study determined that potato and yam agar were the most suitable nutritional sources for the high biomass cultivation of *P*. *ostreatus*. Other studies have utilized potato dextrose agar, malt extract agar, glucose peptone agar, yeast malt agar, saboraud's dextrose agar, and czapex dox agar to determine the extent and efficacy of *Pleurotus* spp. cultivation (Fletcher, 2019; Kirsch et al., 2016; Kumar et al., 2018; Nguyen & Ranamukhaarachchi, 2020). On the other hand, Soni et al. (2018) utilized a combination of carrot powder, rice, and pea protein concentrate medium for the cultivation of *Pleurotus* sp.

Nitrogen, a significant factor in the production of enzymes, is required for protein, nucleic acid, purine, pyrimidine, and polysaccharide production constituents of the cell wall of fungi (Bellettini et al., 2019; Pereima & Ivanova, 2017). Various concentrations of organic and inorganic nitrogen sources have been included in culture media. Organic sources include amino acids, beef extract, hydrolyzed proteins, tryptic soy broth, malt extract, yeast extract, and urea. Whereas inorganic sources include ammonium acetate, ammonium chloride, ammonium nitrate, ammonium phosphate dibasic, ammonium sulfate, ammonium tartrate, potassium nitrate, and sodium nitrate (Deshaware et al., 2021; Kirsch et al., 2016; Krakowska et al., 2016; Zhang et al., 2023). Therefore, establishing a suitable culture media as a nutritional and/or nitrogen source in the laboratory is of the utmost importance, which may not be restricted by the high cost of culture media components.

#### 3.1.3 Agro‐industrial biowaste‐based media

Food waste integrates three major concerns: food security, greenhouse gas emissions in the food supply chain, and waste disposal (Cronjé et al., 2018). Increased production of fruits and vegetables has resulted in massive waste increment that includes inedible components such as roots, skins, seeds, kernels, peels, shells, and leaves that must be discarded (Magama et al., 2022). The composition of waste is critical because it influences the extent to which the waste meets the criteria for usage in multiple processes. This includes a high concentration of biodegradable organic compounds that are high in carbohydrates, starch, cellulose, soluble sugars, minerals, and organic acids. Proteins, lipids, fiber, and other nutrients such as vitamins and minerals are also abundant (Magama et al., 2022).

Since *Pleurotus* spp. mushrooms grow on wood in nature, usually on dead, standing trees, or on fallen logs, therefore various substrates (such as maize, wheat, rice straw, cotton stalks, or waste hulls) containing lignin and cellulose, can be utilized in culture medium for mycelium production (Cohen et al., 2002). As fungi secrete enzymes that digest the lignocellulosic materials to support mycelial growth, they can effectively recycle agricultural waste (Lu et al., 2020). Studies have described the growth of oysters, wild mushrooms and mycelia using waste residues from coconut shells (Bermúdez et al., 2001), peat (Hong et al., 2012), pumpkin and papaya peels (Behera & Gupta, 2015), cucumber and onion (Shashitha & Singh, 2016), pineapple skin and rice washing water (Mujdalipah & Putri, 2020), as well as the use of fruit and vegetable waste hydrolysates as a microalgae cultivation growth medium (Magama et al., 2022). By cultivating mycelia on inexpensive substrates and media, its usage in various powdered and tablet forms as dietary supplements or as components of functional foods (Krupodorova & Barshteyn, 2015) has gained interest recently.

The cultivation of mycelia using diverse culture media, including optimized carbon, nutrient, and nitrogen sources as well as biowaste‐based media, not only enhances the growth efficiency and bioactive compound production, but also aligns with sustainable practices, offering a promising avenue for future applications in biotechnology and food industries.

### 3.2 Mycoprotein as an alternative protein source

Proteins are the most essential functional component of many plant‐based foods due to their distinctive structuring, texturizing, emulsifying, foaming, fluid retaining, and nutritive properties (McClements & Grossmann, 2021). Nonmeat protein or plant‐derived proteins are defined as those having zero servings of dairy, meat, poultry, fish, and eggs. However, according to an analysis of ‘‘plant‐based’’ foods in the large US National Health and Nutrition Examination Survey (NHANES), it provides servings of fruits, vegetables, legumes, grains, soy products, and nuts/seeds (Derbyshire, 2020).

Mycelial protein levels have been reported to compete positively with those of leguminous plant‐based replacements, such as peas, chickpeas, soy, or lupine (Maini Rekdal et al., 2024). Whereby extraction, precipitation, and centrifugation are popular methods that have been reported for isolating proteins from disintegrating mycelia (Berger et al., 2022). Soybeans, wheat, peas, and lupine contain protein content ranging from 5% to 50% on dry weight basis (dwb.) (Smetana et al., 2023). Fungi, on the other hand, can produce significant amounts of protein (ranging from 20% to 85%, dwb.) depending on the genus, species, and culture conditions (Bakratsas et al., 2021; Whittaker et al., 2020).

Mycoproteins (dried biomass of fungi) or fungal proteins are alternative proteins derived from the cultivation processes of fungi in mycelial biomass. Although cultured mycelia are not normally consumed, they can be used as food ingredients and flavoring agents. They are also regarded as alternative protein sources that can be used for human or animal nutrition (Lu et al., 2020; Stoffel et al., 2019). Mycoprotein has been reported to contain all the essential amino acids and inorganic components such as iron, zinc, salt, selenium, manganese, calcium, phosphorus, and vitamin B_2_ (Ahmad et al., 2022). When compared to milk, mycoprotein has a higher net protein utilization value (Ahmad et al., 2022).

Mycoprotein was discovered in the 1960s after being derived from a naturally occurring filamentous fungus *Fusarium venenatum*. Over the last three decades, fermentation processes have successfully produced mycoprotein (Derbyshire, 2020). Although *F. venenatum* is the basis for meat substitutes marketed under the brand name Quorn, its manufacture is intensive and costly (resources and energy utilization). The fungus is generated in bioreactors by a continuous fermentation process. Following fermentation, the ribonucleic acid (RNA) must be degraded into monomers, and the residual biomass is processed to produce a product with a solid content of 20%. To obtain fibrous products, further processes such as forming, steaming, chilling, and texturizing are required (Dekkers et al., 2018).

Researchers have attempted to develop novel alternative technologies that might utilize low‐cost substrates to keep prices low and profit margins high. Date waste, pea‐industry by‐products, brewer spent grain, grape bagasse, cheese whey, and various industrial wastewaters (microalgae/purple bacteria) have all been used to extract mycoprotein (Ahmad et al., 2022). According to a study by Souza Filho et al. (2018), the pea‐processing byproduct was shown to be an efficient medium for the growth of a biomass filamentous fungi to produce a vegan‐protein concentrate (between 46% and 54% protein content). The bakery and confectionery industry are also being challenged to develop products with improved physical–chemical, sensory, and nutritional attributes, due to the increased customer demand for nutritious, high‐quality food. A study by Stoffel et al. (2021) determined that using *Pleurotus* sp. mycoprotein flour increased the nutritional value of the cookies by providing more protein, dietary fiber, an increase in phenolic content, and an increase in antioxidant activity when compared to cookies formulated using wheat flour. For Stoffel et al.’s (2019) study, brewer‐spent grain and grape bagasse were utilized in the solid‐state cultivation of mycelial biomass to produce mycoprotein flour. This was a promising technique for nutritional enrichment and the generation of bioactive compounds with the potential to become a functional food product. Nonetheless, mycoprotein used in the production of meat alternatives has its benefits and limitations, which are outlined in Table 3 below.

**TABLE 3:** The benefits and limitations of mycoprotein utilization.

Category	Benefits	Limitations	References
**Nutritional composition**	▪ Complete protein due to availability of all essential amino acids. ▪ Higher protein (41% dwb.) content than commonly consumed plant‐based proteins. ▪ Low in saturated fat and cholesterol. ▪ High in dietary fiber and easily digestible. ▪ Suitable option for vegans and vegetarians.	▪ It lacks animal‐based protein nutrients such as iron, omega‐3 fatty acids, and vitamin B_12_. ▪ Consumers may associate vegetable oils used in culture media as processed or less natural products.	Souza Filho et al. (2018), Finnigan et al. (2019), Romão et al. (2023)
**Environmental sustainability**	▪ Cultivated using fewer resources (land and water). ▪ Has a low carbon footprint and generates fewer green‐house gas emissions. ▪ Cultivation and production can be easily changed according to demand.	▪ Higher energy consumption (techno economics) due to medium cultivation.	Smetana et al. (2015), Finnigan et al. (2019)
**Processing and additives**	▪ Reduce reliance on animal products. ▪ Combining it with other plant‐based proteins can help it achieve a more balanced amino acid profile. ▪ It is versatile and flavorful (umami), which can be used to resemble various meat‐based products.	▪ The processing to improve flavor, texture, and shelf‐life may involve the use of additives, flavorings, and preservatives. ▪ The incorporation of methylcellulose and various gums during processing is subject to regulatory safety and food standards.	Smetana et al. (2015), Romão et al. (2023)
**Side effects**	▪ Incidence of allergic reactions to mycoprotein remain exceptionally low, but people are less sensitive to it than they are to soy and eggs.	▪ Existing allergies to fungal‐based products. ▪ Excessive consumption may cause bloating or upset stomach due to high chitin and glucan (fiber) levels.	Finnigan et al. (2019), Stoffel et al. (2021)

Dry weight basis—dwb.

Consequently, the development of mycoprotein enables the production of sustainable high‐quality proteins, including essential amino acids that can be utilized as meal components in line with current dietary guidelines (Majumder et al., 2024). According to research, its significance as a dietary component can support metabolic health and maintain protein synthesis rates like those reported in omnivorous diets (Derbyshire et al., 2023).

## 4 CELLULAR AGRICULTURE: FUTURE PROSPECTS

An emerging technology, cellular agriculture enables the development of animal‐derived agricultural products from cells in a bioreactor as opposed to processing them directly from animals (Mattick, 2018). Cellular agriculture has the potential to be a sustainable and environmentally friendly method of producing alternative meat and meat products since it can be obtained efficiently without the need to develop other supporting tissues and functional structures such as skeletal and digestive systems (Zhang et al., 2020). Animal meat, or skeletal muscle is known to comprise approximately 90% muscle fibers, 10% connective and fat tissues, and 0.3% blood. Additionally, it encompasses several flavor molecules such as amino acids, hemoproteins, sulphur and carbonyl compounds, lipids, short peptides, and other flavor volatiles (Ben‐Arye & Levenberg, 2019). Nonetheless, emerging engineering approaches have enabled the production of some of these profiles in vitro, by the development of several culture methods (Mattick et al., 2015). Multiple technologies, including cellular agriculture, can be investigated for imitating the desired texture of traditional meat products classified as ground meat, comminuted processed meat, or whole‐muscle meat (Dekkers et al., 2018). The concept of cellular agriculture also covers the development of plant products with little and/or no plant involvement, with the purpose of meeting potential demands for food and nutrition (Nyika et al., 2021).

### 4.1 Categories of cellular agriculture

Cellular agriculture can be categorized into two groups depending on the technology used. (a) Tissue engineering‐based/cellular agriculture entails obtaining live animal cells and culturing them to control cell proliferation and differentiation to direct the formation of increasing quantities of a desired cell type (e.g., muscle and fat for meat) (Boukid et al., 2022; Singh et al., 2022; Stephens & Ellis, 2020). Cultivated meat is engineered so that it is composed of the same cell types (cell products) that are the same or a comparable structure as animal tissues, thus replicating the sensory and nutritional characteristics of conventional meat (Rubio et al., 2020). In general, stem cells with the ability to self‐renew and differentiate are extracted from an animal biopsy, expanded, and differentiated in the laboratory to form muscle fibers, fat, or other cell types that comprise muscle tissue. Depending on the separated cell type and desired characteristics of the final product, these cells are collected and stimulated to generate edible meat end‐products (Guan et al., 2021; Post et al., 2020).

(b) Fermentation‐based cellular agriculture genetically modifies bacteria, yeast, or algae with or without recombinant DNA so that when fermented in glucose (primary substrate) they produce organic molecules. Which can be processed to biofabricate familiar products that include milk, cheese, egg whites, leather, and meat components (scaffolds) (Boukid et al., 2022; Singh et al., 2022). It produces alternative sources of amino acids and peptides, which include biomass using fermentative cultivation of fungi or other bacterial cultures (e.g., algae). Fermentation, also known as acellular production, is the most efficient method of producing amino acids. The process can also provide inexpensive sources of enhanced amino acids, lipids, flavors, additives, vitamins, and minerals (Post et al., 2020).

### 4.2 Cell‐cultured meat

Cell‐cultured meat, an aspect of the final stage of cellular agriculture, focuses on the production of meat products. It is the emerging field of cultured meat, commonly referred to as “clean meat,” “cultivated meat,” “cellular meat,” “in‐vitro meat,” or “lab‐grown meat.” It competes with and works together with advances in plant‐based proteins (Stephens et al., 2018), which create products that are molecularly identical to traditional meat but produced through bioprocesses from animal cells isolated through biopsies (unharmed animal) (Newton & Blaustein‐Rejto, 2021).

Cells can be replicated using a cell culture methodology that includes serum‐supplemented media containing all the nutrients required for cell growth, such as amino acids, lipids, vitamins, and salts (Dekkers et al., 2018). A culture medium without antibiotics and animal components is required for both cell expansion and differentiation, irrespective of which stem cell type is used. These primary cell lines include stem cells (embryonic stem cells and induced pluripotent stem cells), satellite cells (myoblasts), fibroblasts, and epithelial cells (Guan et al., 2021; Post et al., 2020; Santo et al., 2020). Amid that, one of the most significant cost drivers during cell line production upscaling has been identified as the cost of cell‐culture medium (Post et al., 2020).

#### 4.2.1 Source of animal cells

There are two methods used for expanding cells; one requires only one animal, while the other requires a continuous stream of animals. Since adult stem cells take about 50–60 times to replicate before they reach their full capacity, they need to be replaced. The industry standard suggests obtaining adult muscle stem cells from a biopsy of a living or dead animal. Additionally, a biopsy is required whenever a new line (end‐product) of meat cell is produced (Santo et al., 2020). Mattick et al. (2015) recommended that the most promising approach begins with the extraction of adult stem cells from a donor animal tissue sample. These stem cells are placed in a culture medium, which allows them to divide and develop in biomass. When a growth cycle (differentiation and tissue development) is over, the cells are removed from the broth for further processing and packaging.

Adult skeletal muscle stem cells (satellite cells) are the most accessible myogenic progenitor in skeletal muscle tissues. Myoblasts, the satellite cell amplifying progeny, require significant optimization to increase their proliferative capacity for adaption to industrial‐scale cultured meat manufacturing applications (Post et al., 2020). Another method involves the collection of unfertilized eggs from female animals and fertilizing in a petri dish using sperm. Generating an embryonic stem cell line allows them to be utilized indefinitely and be manipulated into muscle fibers. However, the meat produced from this technology would have to be labelled as a genetically modified organism (GMO) and could undergo genetic modifications that might lead to safety concerns or logistical challenges (Santo et al., 2020).

Alternatively, 10‐day‐old, fertilized chicken embryonic eggs (pathogen‐free) can be collected and used for cell isolation. In which the embryo is macerated and used indefinitely by manipulating into muscle fibers (fibroblasts) (Hernandez & Brown, 2010). The benefits of utilizing embryonic stem cells are that they are a type of stem cell that can develop into any tissue (Post et al., 2020).

### 4.3 Current trends on cellular agriculture

Cellular agriculture further promises a broad range of cultured products, from beef to egg whites, as well as substantial food products containing the same proteins as traditional meat products. Recently, critical analysis and the pilot testing of alternative meat products have demonstrated a very compelling alternative for obtaining protein‐rich and nutritionally balanced food materials (Helliwell & Burton, 2021). Professor Mark Post, Chief Security Officer of Mosa Meat in the Netherlands, unveiled the first slaughter‐free hamburger based on laboratory‐cultured beef in 2013 (Rischer et al., 2020). In South Africa, Mogale meat company (rebranded as WildBio) developed a prototype for a cell‐based chicken breast composed of real chicken muscle and fat cells blended with a mushroom matrix, in 2022 (Ferrer, 2022). Additionally, Mzansi Meat was the first company to present its cultivated beef burger at an event in 2022 (Tsvakirai et al., 2023). Seeing as ground beef is notably easier to replicate than steak, three‐dimensional (3D) printing has also been considered as one of the solutions for recreating a steak made from cultured beef (Rischer et al., 2020).

When considering food security challenges on the conservation of species, a centralized model of cellular agriculture would not address the biosafety danger posed by wildlife markets. The elite's demand for exotic or rare products is frequently attributed to the illegal poaching of endangered species, with several illegal hunting driven by the cultural significance of the animal product to the local population (Soice & Johnston, 2021). Given that, Tuomisto and Teixeira de Mattos (2011) emphasized the development of wildlife‐based cultured meat that would give the market an ecologically responsible product, essentially contributing to the conservation of native animals and alleviating sustainability issues. The goal is to either reduce or slow the expansion of usage of animals in agriculture worldwide to meet the global rising demand for animal products compelled by population and wealth increases (Stephens & Ellis, 2020).

The effects and risks of global warming on oceans threatening to devastate wild fish populations offer cellular agriculture of seafood a once‐in‐a‐lifetime potential to make radical changes in the food system. Research by Rubio et al. (2019) on the production of seafood from marine cell cultures displays a revolutionary seafood production method and an attractive possibility for cellular agriculture. At the same time, consumer acceptance is a fundamental driver of success in food‐product development, which still remains unsolved. Carneiro et al. (2022) review summarized information on 31 fish flavors, outlining current flavor development challenges as well as prospective uses on 32 cell‐cultured fish products. Consequently, emphasizing the usage of salts, natural antioxidants, clever packaging, and other processing techniques to achieve cell‐cultured fish with a salty umami taste and sea‐like flavors.

Decisively, these reviews on literature provide comprehensive overviews of the current state of research, identifying key findings and knowledge gaps, thus offering valuable educational resources for researchers and professionals alike. Insight on the opportunities and challenges (Böl et al., 2021), environmental implications (Mattick et al., 2015), progress and prospects (Nyika et al., 2021), attitudes toward cultured meat and nutrition (Finnigan et al., 2019; Ismail et al., 2020), and consumer acceptance (Pakseresht et al., 2022) are crucial in advancing cellular agriculture technologies.

On the other hand, surveys often reveal the public's perception and attitude toward cellular agriculture, which may vary among different demographic groups, including age, gender, income, and ethnicity. Also providing essential information on the attitudes of potential consumers and factors affecting consumer acceptance in various countries (Chezan et al., 2022; Pakseresht et al., 2022; Palmieri et al., 2020; Szejda et al., 2021; Wilks & Phillips, 2017). Consumer surveys on cellular agriculture are significant in supplying essential data to companies, researchers, policymakers, and advocacy groups working in this industry, as well as playing an important role in identifying and addressing consumer concerns and preferences (Moritz et al., 2022).

The adoption of cellular agriculture's final products is still moderate, with cellular food processing being undertaken in small amounts, except that the challenge of largescale and cost‐efficient manufacturing remains uncertain (Nyika et al., 2021). Grasso's (2024) viewpoint explored the pricing and production capacity of hybrid meat products, as well as their potential for scalability. The article emphasized that long‐term practical feasibility is uncertain and hinges on overcoming challenges related to customer awareness, pricing, marketing, and product development, while also leavening opportunities such as hybrid meals and appealing to flexitarian consumers. This highlights the crucial role of effective communication and reaching a broad audience to boost sales and support larger production runs. Additional challenges draw attention to how critical it is to give regulatory procedures and the name of cultured products careful consideration, since these steps are necessary to ensure that the products are consumer‐friendly, reflect scientific methods, and comply with regulations. In 2023, the World Health Organization (WHO) and the Food and Agriculture Organization of the United Nations (FAO) are now adopting the phrase “cell‐based food” as a working terminology; however, this term has not yet been officially harmonized (WHO, 2023).

Nonetheless, certain start‐up companies in the field of cellular agriculture have been granted financial support, providing significant capital for research and development. This has been observed in nonprofit organizations and foundations (e.g., The Good Food Institute and Cellular Agriculture Society) that are dedicated to funding research and development in sustainable agriculture and food production (Stephens et al., 2018). Numerous competitions and innovation challenges (e.g., XPRIZE Feed the Next Billion and FoodShot Global) have provided groups involved in cell‐cultural processes with cash prizes and resources (Telesetsky, 2023).

### 4.4 Hybrid cell‐cultured meat analogues

Meat analog products have been traditionally targeted toward vegetarians and vegans. Despite this, given consumers' increased perception and knowledge of health and environmental concerns, meat‐eating consumers are also presented with these options (Chandler & McSweeney, 2022). Several cultured products are being researched and produced, receiving considerable attention from social scientists (McClements et al., 2019).

Hybrid cell‐cultured meat products are a hybridization from a combination of animal‐based, plant‐based, and fermentation‐based proteins derived from cultured animal cells, sources of plant‐based ingredients, and/or fungi‐based ingredients (Lee et al., 2022). This combination is developed and manufactured using functional ingredients listed on Table 4, which resemble the taste, texture, nutritional composition, and appearance of conventional meat. A combination of these ingredients may vary according to the desired characteristics of the final product, thus improving their overall composition. Whereby to produce a hybrid meat product, plant‐based components are reported to be included in quantities ranging from 20% to 50% (van Dijk et al., 2023).

**TABLE 4:** The functionality of various ingredients used in the formulation of assorted meat analogs.

Functionality	Examples of ingredients	Significance in formulation	References
**Coloring**	Red meat color: apple extract, annatto extract, beet juice extract, betanin, soy leghemoglobin, tomato paste. Chicken color: titanium dioxide, fermented rice flour, paprika oleoresins.	▪ Improves the visual appearance of the product. ▪ Used to differentiate the various types of products. ▪ Mimics the appearance of specific parts of meat. ▪ Result in the browning reaction during cooking.	Lee et al. (2020), Boukid (2021), Kyriakopoulou et al. (2021)
**Emulsifier**	Calcium phosphate, chia mucilage flour, chestnut flour, chickpea‐protein, mung bean protein, prolamin‐based zein/corn fiber gum, pea protein isolate, sunflower lecithin.	▪ Stabilization and prevent separation of oil and water phases during processing and cooking. ▪ Stabilizes oil‐in‐water Pickering emulsions. ▪ Used to replace or reduce the amount of fat incorporated.	Cabezas et al. (2016) Song et al. (2021)
**Fat replacer**	Canola oil, chia oil, coconut oil, flaxseed oil, hemp oil, linseed oil, olive oil, palm oil, sesame oil, soybean oil, sunflower oil, tiger nut oil.	▪ Improves the flavor, texture, or mouthfeel of the product. ▪ Retains the moisture content of the product. ▪ Responsible for marbling, mimicking appearance of fat.	Botella‐Martinez et al. (2022)
**Flavor and seasoning**	Beef‐like aromatic substances (thiol, pyrazines, thiazoles, disulphides), cultured dextrose, herbs, paprika, salt, spices, sugar, savory yeast extract.	▪ Enhances the flavor profile of the product. ▪ Produces desirable flavor compounds. ▪ Mask off‐flavors, astringent, pungent aromas, beany, and bitter notes.	Zhang et al. (2020), Boukid (2021)
**Gelling and/or binding agent**	Agar, alginate gel, carrageenan, casein, gelatine, guar gum, konjac gum, pectin, soy protein isolate, inulin, locust bean gum, methylcellulose, modified starches, mung bean protein isolate, potato starch, prolamin‐based zein, whey protein isolate, xanthan gum.	▪ Retains or binds water, contributing to cohesiveness and resilience, forming a meat‐like structure. ▪ Replicates the mouthfeel and consistency of the product. ▪ Improves the moisture retention of the product. ▪ They can determine the production processing conditions of the product. ▪ Replace or substitute fat in burgers, sausages, or nuggets.	Boukid (2021), Ng and Kurisawa (2021), Song et al. (2021), Botella‐Martinez et al. (2022)
**Texturizing**	Textured soybean, pea protein, mung bean protein, mushroom mycelium, oat flour, wheat gluten.	▪ Improves the texture modification of the product, by binding to water and being a source of insoluble fiber. ▪ Mimics the fibrous and chewy texture of muscle fibers in meat. ▪ Improves the nutritional profile and protein content, including essential amino acids in the product. ▪ 3D scaffold material is used to support cells (structure).	Ben‐Arye et al. (2020), Maningat et al. (2022), Flores et al. (2023)
**Nutritional enhancement**	Calcium phosphate, potassium phosphate, sodium ascorbate, sodium phosphate, thiamine hydrochloride, tocopherols, zinc gluconate.	▪ Contribute to the overall nutritional profile of the product. ▪ Improve protein solubilization. ▪ Replicate the composition of conventional meat, to achieve the recommended daily allowance.	Boukid (2021), Molina et al. (2023)

Muscle fiber maturation and alignment, extracellular matrix protein content and alignment, intramuscular fat content, and structure are all factors that might affect meat texture (Ben‐Arye & Levenberg, 2019). Meat substitutes created involving a variety of protein texturizing processes have been intensively studied throughout the years. The idea was initially created in the 1970s to create a texturizing method employing a variety of plant‐based ingredients, primarily soy (Zahari et al., 2022). These various plant‐based texturizing ingredients and proteins have been included in formulations.

Mycelia, on the other hand, are composed of fibrous carbohydrates, which can successfully mimic the texture of muscle meat, better than plant‐based proteins (Kumar et al., 2017). However, as mycelia solely cannot form strong gels in meat formulations, a study by Santhapur et al. (2024) demonstrated that potato proteins (containing 90.5% protein) can form robust heat‐set hybrid gels due to their protein molecules when combined with mycoproteins. These hybrid gels are useful for developing and producing new plant‐mycoprotein hybrids, as their mechanical strength surpasses that of potato protein gels, attributable to the mycelia fibers serving as active fillers within the potato protein network.

Mycelium texture can alternatively be modified further through pressing, extrusion, or other mechanical techniques (Kumar et al., 2017). Mycelium with a meat‐like (umami) flavor can also be a novel dietary source for meat analogs. Given that mycelia have a high glutamic acid and sulphur‐containing amino acid content, it can substantially improve the umami flavor and taste of meat analogs (Barzee et al., 2021; Kumar et al., 2017). Tagkouli et al. (2021) observed that roasting mushrooms (*P*. *eryngii* and *P*. *ostreatus* strains) led to the formation of sulphur compounds that enrich food flavor with meat‐ or roast‐like flavors. Given the limited research on incorporating mushroom products in meat analogs, this information is particularly relevant. The selection of fungal species is a significant factor in determining the flavor of the fungi‐based product. Owing to the culture medium and culture period impacting the mycelial biomass, which may or may not be desirable for certain product formulations. On the other hand, less naturally flavored mycelia can be cultivated in media supplemented with spices, herbs, and extracts to impact the flavor aspect of the final product (Barzee et al., 2021).

Blending mycelia with plant‐based pulses (such as soy or pea) could enhance the fibrous texture and protein content, but a significant challenge is the presence of off‐flavors, which give beany aromas in meat analogues, resulting in consumer dissatisfaction. This was demonstrated in a study by Barker and McSweeney (2022), which utilized yellow pea flour and chicken to create a hybrid meat burger to assess its sensory characteristics. Increasing the proportion of pea flour in the formulation lowered the overall protein content, introduced beany off‐flavors, and resulted in undesirable textural properties, ultimately reducing consumers preference. Flores et al. (2023) found that these off‐flavor volatile compounds can be removed by deodorization using ethanol as a solvent. While this deodorization process proved effective, it altered the textural properties by solubilizing the proteins, thereby reducing their availability and ability to form a network, ultimately limiting their functionality in hybrid meat patties.

When formulating cultured meat researchers also have recommended that meat analogs feature color characteristics comparable to conventional raw or cooked meat. Considering that raw meat has a bright red color due to high oxymyoglobin levels, the flesh color changes to brown and metmyoglobin content increases when cooked (Lee et al., 2020). Additionally, consumers seek food‐grade ingredients that are nontoxic and more natural, and researchers have recommended various ingredients, coloring agents, and substances that can impact these similar properties or enhance that specific animal flavor profile (Table 3). Olewnik‐Mikołajewska et al. (2024) study introduced hybrid meat sausages incorporating cereals ingredients such as rice, wheat, oats, which demonstrated enhanced nutritional value and higher health benefits compared conventional meat sausages. They developed two type of ready‐to‐eat cabanossi sausages: one with groats and another with sunflower seeds. The groat‐based sausages (containing 70 g of chicken meat per 100 g of product) exhibited lower fat and energy content compared to the sunflower seed‐based sausages (containing 75 g of chicken meat per 100 g of product). Despite the variations in physicochemical properties, both types were found to be acceptable from a sensory perspective.

Furthermore, by including meat analogues generated from cell cultures using fibroblasts (for firmness) and fat cells with myotubes (for taste), hybrid meat analogs with a meat‐like appearance and texture could also be created (Bhat et al., 2020; Rubio et al., 2020). The limitations of cell culture procedures in the production of meat components are that they lack the consistency, vascularization, or fat‐marbling components of traditional meat. By generating organs for transplantation procedures, 3D structures can address these issues by employing the principles of standard printing technology. Using spraying solutions of single cells or balls of cells onto gels that act as printers, meat structure and shape can be mimicked efficiently (Bhat et al., 2017). Wang et al. (2022) explored this 3D printing process to create textured, soft hybrid meat analogs incorporating finely minced chicken, pea protein isolate, maize starch, beef fat, and soy lecithin. The effectiveness of the printing process was influenced by factors such as nozzle size, printing speed, and infill density. They were successful in creating a 3D printed meat analog with 20% chicken using a 1.54‐mm nozzle size, but faced challenges as the shape was slightly damaged after boiling in water and highlighted the need to increase printing speed for industrial applications. This innovative technology not only enhances the nutritional value and sensory qualities of meat analogs, but with improved formulations, it could also provide better texture, making it more palatable for the elderly with aging constraints as proposed by Wang et al. (2022).

While it is easy to examine the various components and processes that constitute a hybrid product, establishing consumer preferences is equally crucial. Baune et al. (2023) conducted an evaluation of the sensorial attributes, consumer acceptance, and nutritional qualities of hybrid meat products, revealing a high consumer willingness to purchase these variants. Both trained and untrained panelists participated in the study, identifying a meatball formulation with 30% processed pea protein as the most acceptable. This product demonstrated superior nutritional score compared to pure meat (control), except for the presence of off‐flavors.

Most consumers are generally unfamiliar with hybrid meat products and have low expectations regarding their taste. Researchers and the food industry must still demonstrate the existence, superiority, quality, and significant nutritional value of these products (Baune et al., 2023). Although some hybrid meat products are often well perceived in terms of taste and texture, this does not necessarily guarantee market success (Grasso, 2024). The technical and sensory aspects (texture, appearance, taste) of producing hybrid meat analogs using fungi‐based and cell‐based components could potentially be seen as both promising and challenging, particularly given to the limitations of plant‐based products. As a result, it is essential to optimize the development of innovative meat alternatives that will model as sustainable, efficient and viable sources while incorporating consumer preferences.

## 5 CONCLUSION AND FUTURE ADVANCEMENTS

Mycelia can be cultivated on a wide range of culture media compositions and types, making this process versatile. The ultimate methods of culturing mycelia will reduce the reliance on mass systems of cultivation, achieving higher yields in a shorter amount of time. Introducing mycoprotein or single‐cell protein (extracted from dried mycelial biomass) into the human diet could be one practical solution for alternative protein products that are reliable and sustainable. Coculturing with various cell types may improve cultured meat quality even further. Hybrid cell‐cultured meat analogs can also be essential as the industry and consumers gradually move toward broader availability of cultivated meat products that may be easier to industrialize. These products can be widely available on the global market, establishing them as the industry standard for vegan or vegetarian protein sources, health‐conscious nonvegetarians, meat substitutes, dietary supplements, and flavor or taste preservatives. However, a significant commercial hurdle is the lack of consumer understanding about what hybrid cell‐cultured meat products are and their benefits. This confusion can prevent consumers from choosing these products over traditional meat options, impacting sales and market feasibility. While plant sources may be cheaper than meat, the cost of processing and incorporating the various ingredients into hybrid products may affect the final product price. High‐production costs can make it challenging to sell these products at a price acceptable to consumers while remaining profitable for manufacturers. In the fast‐paced retail environment, products must meet sales expectations and a consistent market demand to remain on the shelves. Therefore, products that target specific consumer needs, offer a balanced meal, or provide added convenience have a higher chance of success. To avoid confusion and ensure fair competition with traditional meat sources, accurate product labelling with the sources and components of ingredients must be clearly listed, complying with existing and emerging regulatory requirements. Technological issues related to the functionality (i.e., emulsifying or gelling ability, water and oil holding capacity) of each ingredient must be considered, as these properties can vary significantly within different batches of the same protein source, making it difficult to achieve consistent results in large‐scale production. Additionally, in the development and optimization of hybrid products, the ratios of plant‐based, fungi‐based, and animal‐based ingredients must be carefully balanced to avoid negative sensory attributes (mainly flavor and texture) that could impact consumer acceptance. The sourcing of plant‐based and animal‐based ingredients, especially cell‐based sources, may be subject to certain regulations and standards (e.g., premarket approval and safety assessments) that can significantly impact the production and commercialization process. Any environmental, sustainability, health claims, or novel food status made about the product must be backed up by scientific evidence, life cycle assessments (LCAs), legal definitions and restrictions, and regulatory bodies. This evolving landscape of cellular agriculture, encompassing diverse categories such as cell‐cultured meat derived from various animal sources, current trends in innovative food products and research practices, and the development of hybrid‐cell‐cultured meat analogs, highlights a promising future for ethical and nutritious food production that is poised to revolutionize the global food industry. It can be said that cellular agriculture and the application of fungal biotechnology, a potentially additional food production method, has the potential to expand the availability of various protein products. Supplementary research and development in the food and agriculture sectors can solve the open problems related to the overall quality, sustainability, and safety of various cellular agriculture approaches on the global market.

## AUTHOR CONTRIBUTIONS

**Kayise Hypercia Maseko**: Conceptualization; investigation; writing—original draft. **Thierry Regnier**: Conceptualization; visualization; writing—review and editing; supervision. **Paul Bartels**: Supervision; writing—review and editing. **Belinda Meiring**: Conceptualization; writing—review and editing; supervision; visualization.

## CONFLICT OF INTEREST STATEMENT

The authors declare that they have no known conflict of interest that is relevant to the content of this article.
